# Emotional exhaustion and burnout among medical professors; a nationwide survey

**DOI:** 10.1186/1472-6920-14-183

**Published:** 2014-09-04

**Authors:** Joeri K Tijdink, Anton CM Vergouwen, Yvo M Smulders

**Affiliations:** Department of Internal Medicine, VU University Medical Centre, PO Box 7057, 1007 MB Amsterdam, The Netherlands; Department of Psychiatry, Tergooi Hospital, Blaricum, the Netherlands; Department of Psychiatry, St. Lucas Andreas Hospital, Amsterdam, the Netherlands

## Abstract

**Background:**

Although job-related burnout and its core feature emotional exhaustion are common among medical professionals and compromise job satisfaction and professional performance, they have never been systematically studied in medical professors, who have central positions in academic medicine.

**Methods:**

We performed an online nationwide survey inviting all 1206 medical professors in The Netherlands to participate. They were asked to fill out the Maslach Burnout Inventory, a ‘professional engagement’ inventory, and to provide demographic and job-specific data.

**Results:**

A total of 437 Professors completed the questionnaire. Nearly one quarter (23.8%) scored above the cut-off used for the definition of emotional exhaustion. Factors related to being in an early career stage (i.e. lower age, fewer years since appointment, having homeliving children, having a relatively low Hirsch index) were significantly associated with higher emotional exhaustion scores. There was a significant inverse correlation between emotional exhaustion and the level of professional engagement.

**Conclusions:**

Early career medical professors have higher scores on emotional exhaustion and may be prone for developing burnout. Based upon this finding, preventive strategies to prevent burnout could be targeted to young professors.

## Background

Burnout is described as ‘a prolonged response to chronic emotional and interpersonal stressors at work’, and is three dimensionally defined by ‘emotional exhaustion’, ‘depersonalisation’, and ‘reduced personal accomplishment [1]. Previous studies suggest that burnout, particularly emotional exhaustion, is common among physicians [2–7], affects morale and productivity, but also reduces quality of care and predisposes to medical errors [8–11].

Reported risk factors for burnout in the general population include being young, single, and childless. As for job-related factors, home-work interface stress and being at the early stage of a professional career appear to increase risk for burnout. In physicians, risk may be aggravated by job-specific circumstances such as demanding patients, reduced resources, and the threat of liability [12–16].

Opposite of burnout stands engagement, defined as ‘a positive, fulfilling, work-related state of mind, characterised by vigour, dedication and absorption’ [17], and it has been suggested that strong professional engagement may protect against burnout [18].

Medical professors are in many ways at the heart of the medical community as they act as, educators, managers and, - perhaps most importantly - role models for students, residents and colleagues. However, these same activities and responsibilities may render them vulnerable to job-related stress and burnout.

To our knowledge, there are some studies evaluating burnout symptoms by academic rank [19, 20] although these symptoms have never been systematically studied in the unique subgroup of medical professors. This study addresses the prevalence, severity and potential determinants of burnout symptoms among medical professors in The Netherlands. Since emotional exhaustion is the core feature of burnout [1, 21–24], the association of emotional exhaustion with personal and job characteristics, with the Hirsch index as a measure of scientific success, and with the level of professional engagement was examined in detail.

## Methods

### Procedure and participants

Professors working at one of the 8 academic medical centres in The Netherlands were sent an invitational e-mail in September 2011 to participate in a survey addressing burnout symptoms, but also aspects of publication culture. We included professors working in either clinical or preclinical disciplines, all being employed by one of the 8 University Medical Centres in The Netherlands.

The e-mail explained the objectives of the study, using neutral terms as ‘work experiences and engagement’, and provided them with a link to an anonymous online questionnaire on a protected website. Those who did not respond were sent a reminder after 3 weeks, and responses were registered until 6 weeks after the first invitation.

### Variables

The questionnaire contained, apart from demographic questions, validated burnout and engagement questionnaires. Burnout was measured using the Dutch version [25] of the Maslach Burnout Inventory (MBI) Human Services Survey [1], which is designed specifically for use in people working in human services and health care. The Dutch version (the Utrechtse Burn Out Schaal (UBOS), see online appendix for English translation) consists of 20 items covering the three domains of burnout: 1] the depletion of emotional reserves (*emotional exhaustion, 8 items*), 2] an increasingly cynical and negative approach towards others (*depersonalization, 5 items*), and 3] a growing feeling of work-related dissatisfaction (*personal accomplishment, 7 items).*

As examples, emotional exhaustion is assessed through questions such as ‘I feel like I am at the end of my rope’ and ‘I feel burned out by my work’, and depersonalization with questions such as ‘I feel I treat some of my faculty and residents as if they were impersonal objects’. Personal accomplishment is assessed with questions such as ‘I have accomplished many worthwhile things in this job’. Items were rated on a 7-point frequency scale (0-6), such that more points on the emotional exhaustion and depersonalisation domain indicated a higher propensity for having burnout). Personal accomplishment is inversely related to burnout: lower scores indicate a higher propensity for having burnout.

Since emotional exhaustion is considered the key component of burnout [1, 21–24], we use emotional exhaustion as the primary outcome measure and main variable to assess burnout.

The nominal cut-off scores for burnout were used. These cut-off levels are sometimes based on the Emotional Exhaustion domain scores only. The Dutch Central Bureau of Statistics , for example, has set the cut-off level for the nominal definition of having burnout on an Emotional Exhaustion subscore threshold of >17.68 points (http://www.cbs.nl, http://www.tno.nl/downloads/Rapport NEA 2010.pdf).

Engagement was measured using the 17-item Utrecht Work Engagement Scale (UWES) [17]. This questionnaire has good psychometric properties [18], and consists of three engagement subscales: vigour (6 items), dedication (5 items) and absorption (6 items). High levels of mental energy and willingness to invest in work define vigour, whereas dedication is defined as ‘feelings of enthusiasm, pride and inspiration’, and absorption implies ‘a sense of time passing quickly and low distraction’. Items were rated on a 7-point Likert scale (0-6). The sum of all items is used as a total engagement score.

The demographic and general background information included gender, age (divided into 5 categories), marital status, having homeliving children, type of specialty; years since appointment (per 5 years), main professional activity (research, education, patient care, or management) and self-reported Hirsch Index, a citation-based individual indicator of scientific impact [26].

In this research no patients were involved; therefore no ethics approval was necessary as the research complies with national regulations (https://www.vcmo.nl/wmo/niet-wmo-plichtig-onderzoek/).

### Statistical analysis

Analysis of Variance was used to compare groups. Pearson’s correlation coefficients were calculated to examine relationships between continuous variables. Multiple linear regression analysis was used to identify independent determinants of burnout scores on a continuous scale. We were cautious to avoid statistical overadjustment with multiple age-related variables. Therefore, we introduced in the first multivariate analysis only demographic and job-specific variables. Variables that conceivably were mediators of effects of demographic and job-specific items were subsequently introduced in a second multiple regression model. In a secondary analysis, logistic regression was performed to analyse the dichotomized burnout scores using cut-off scores. The Statistical Package for the Social Sciences (SPSS) statistics (Chicago USA 2011, version 20) was used for the statistical analyses.

## Results

### Demographics

Of the 1366 e-mail addresses used, 160 bounced, most often because the addresses no longer existed, or repeatedly provided an out-of-office reply. To the remaining 1206 e-mails; 578 professors responded (49%), of whom 437 (36%) completed the full questionnaire. Data on demographic and job-specific characteristics of complete respondents are summarized in Table 1.

**Table 1:** **Demographic and job-specific characteristics of 437 respondents**

		N = 437
Gender	Male	345 (79%)
	Female	92 (21%)
Age	26-35	1 (0,2%)
	36-45	35 (8%)
	46-55	206 (47%)
	56-65	190 (44%)
	65 and older	5 (1%)
Marital status	Married or cohabiting	401 (92%)
	Single	36 (8%)
Home living children	None	217 (50%)
	1	56 (13%)
	2	96 (22%)
	3 or more	68 (15%)
Years since appointment	0-5	150 (34%)
	6-10	129 (30%)
	11-15	86 (20%)
	16 or more	72 (16%)
Nr. 1 Work priority	Research	255 (59%)
	Education	40 (9%)
	Patient care	63 (14%)
	Management	79 (18%)
Appointment	Temporary	144 (33%)
	Permanent	293 (67%)
Raw scores of burn out dimensions	Emotional Exhaustion	11,9 (SD 8,9)
	Total Score (0-48)	
	Depersonalisation	4,4 (SD 4.4)
	Total score (0-30)	
	Personal accomplishment	30,9 (SD 5,9)
	Total score (0-42)	
Raw scores of engagement dimensions	Vitality Total Score (0-36)	28.1 (SD 5.0)
	Dedication Total Score (0-36)	24.9 (SD 4.2)
	Absorption Total Score (0-36)	26.4 (SD 5,4)
Specialty	Preclinical	81% (354)
	Clinical	16% (70)
	Anonymous	3% 913)

### Early-career professors show higher emotional exhaustion scores

Univariate determinants of burnout and engagement (sub) scores on a continuous scale are shown in Table 2. Younger age, less years since appointment, and having children living at home were significantly associated with emotional exhaustion and with at least one other component score of burnout. In the multivariate analysis of demographic and job-specific items, age, home-living children and years since appointment were included, and the latter appeared to be the main, independent age-related determinant of emotional exhaustion (Table 3). We also performed multivariate analysis for depersonalisation in which no effect was found (data not shown).

**Table 2:** **Univariate regression analysis comparing independent variables with burnout and engagement component scores (Vigour, Dedication and Absorption)**

	Burnout domain scores			Engagement domain scores		
	Emotional exhaustion	Depersonalisation	Personal accomplishment	Vigour	Dedication	Absorption
Age (per 10 years	-1.7**	-0.8**	0.3	0.4	-0.1	-0.6
Gender (female)	0.1	0.5	-0.3	-0.4	-0.3	0.7
Marital status (single)	-1.7	-0.3	1.4	0.7	0.7	0.9
Homeliving children (yes)	2.5**	1.1**	0.6	0.9*	0.5	1.2**
Fixed position (yes)	-1.3	-0.0	1.1*	0.7	0.1	0.2
Years since appointment (per 5 years)	-1.3**	-0.2	0.4*	0.4*	0.2	0.0

Regression coefficients are shown and (borderline) significant values are shown by markation: *0.05 < p < 0.10; **p < 0.05. Determinants with a univariate p-value of <0.10 were entered in the multiple regression analyses.

**Table 3:** **Multivariate regression analysis comparing independent variables with emotional exhaustion**

	Beta (95% CI)	p-value
Emotional Exhaustion (0-45)		
Age (per 10 years)	-0.3 (-1.9 to 1.3)	0.72
Homeliving children (yes)	1,6 (-0.4 to 3.5)	0.11
**Years since appointment (per 5 years)**	**-1.0 (-1.9 to -0.1)**	**0.03**

Regression coefficients are shown and significant values are shown in bold (p < 0.05).

According to the aforementioned cut-off level on the Emotional Exhaustion scale, 23.8% of medical professors (n = 104) suffered from burnout.

Logistic regression analysis with the nominal burnout outcome variable identified the same determinants as did linear regression analysis for the continuous subscores, albeit with lower levels of statistical significance (data not shown).

### The role of the Hirsch index

**Figure 1:**
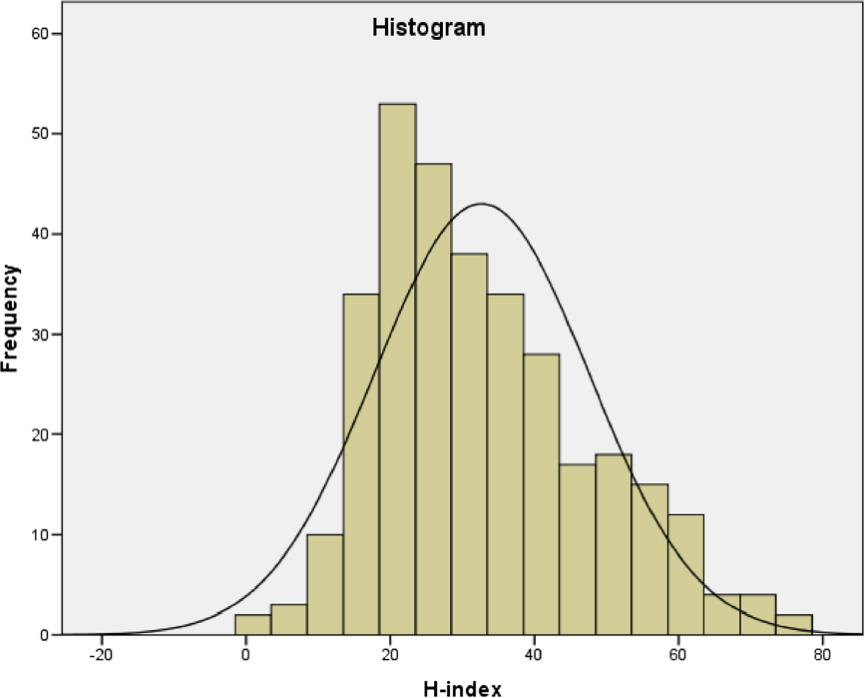
**The distribution of the H-index.**

**Figure 2:**
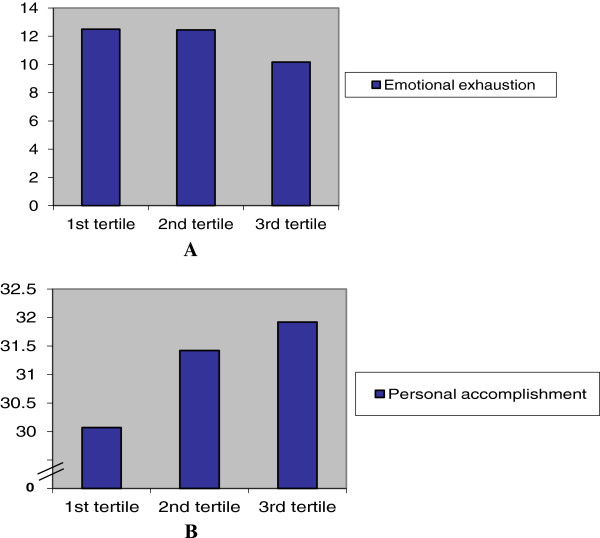
**Score on emotional exhaustion (panel A) and personal accomplishment (panel B), divided in tertiles in h-index score.** 1^st^ tertile h-index ranging from 10-23, 2^nd^ tertile from 24-37, third tertile ranging from 38-78).

Among respondents, 74% knew their current Hirsch index (n = 321), and their average index value was 32.6 (standard deviation: 14.9, see Figure 1 for distribution). The Hirsch index was inversely correlated with burnout symptoms, predominantly with the components emotional exhaustion and personal accomplishment, but not with depersonalization. The highest Hirsch index tertile was associated with a significant 19% lower emotional exhaustion score compared to the lower 2 tertiles (Figure 2, panel A). Personal Accomplishment subscore was significantly and more linearly related with the Hirsch index (Figure 2, panel B, beta per tertile 0.1, CI 0.2-1.7). As the H-index is driven by age, these associations were adjusted for age, which did not change the results. To determine whether the Hirsch index (partly) explains the association between being an early career professor and higher burnout scores on the emotional exhaustion domain, multiple regression was performed (Table 4), suggesting that this was not the case (beta per 5-years since professorship changed from -1.3 to -1.5). Also, no statistical interaction between being early career and the Hirsch index was noted (p = 0.8 for the interaction term).

**Table 4:** **Crude and multivariate analysis of emotional exhaustion including the Hirsh-index as additional independent variable**

	Beta (CI 95%)	p-value
Emotional Exhaustion (0-45)		
Crude analysis		
Years since appointment (per 5 years)	-1.3 (-2.1 to -0.6)	<0.01
Hirsch index (upper vs lower 2 tertiles)	-2.3 (-4.4 to -0.3)	0.02
Multivariate analysis		
Years since appointment (per 5 years)	-1.5 (-2.5 to -0.6)	<0.01
Hirsch index (upper vs lower 2 tertiles)	-1.2 (-3.3 to 1.0)	0.28

### Burn out and engagement

Vigour and dedication were negatively associated with emotional exhaustion (correlation coefficient -0,36 and -0,38, respectively, both p < 0,001), and to depersonalisation (-0,27 and -0,35, respectively, both p < 0,001). All three subscales of engagement (vigour, dedication and absorption) were positively and strongly related to personal accomplishment (0,61, 0,56 and 0,45, respectively, all p < 0,001).

Furthermore, all three engagement subscales showed significant relations with the Hirsch-index (in tertiles, beta’s (CI) 1.1 (0.5 to 1.7), 0.8 ( 0.3 to 1.2), and 0.9 (0.2 to 1.5), respectively, all p < 0.01).

## Discussion

This study suggests that emotional exhaustion is frequent among medical professors, and that the early career years represent a risk period for emotional exhaustion. Having reached a certain degree of scientific success, as indicated by a high Hirsch factor, may confer some degree of protection.

### Interpretation of results

In comparable studies, high burnout frequencies were found in academic chairs in specific medical fields such as gynaecology and orthopaedic surgery [3, 6]. In these studies, 75% of orthopaedic surgeons had moderate to high levels of emotional exhaustion and 54% of gynaecologists reported high levels of burnout (these studies were using different cut-off values compared to this study). We found no previous study addressing an entire nationwide population of medical professors.

Higher emotional exhaustion subscores are found among younger professors, who usually are at the start of their career, and more often have children living at home (not after multivariate analysis). This is in line with previous studies, which found high emotional exhaustion in younger chairs and those with a spouse and children.

Burnout was also more common in new professors [12–15]. These three determinants have a high degree of co-linearity, and may be in each other’s causal pathway. Therefore, the multivariate analysis, which demonstrated that the number of years since appointment is the prime, independent determinant of burnout symptomatology, should be interpreted with some caution. Feeling of control over work and spouse support are two important protective factors against burnout. Effects of seniority may be explained via these effects, since professional experience may increase the (sense of) control over work and work hours [27]. A possible survivor bias is conceivable but not very likely since professors leaving their position in their early years are very rare.

We analysed the potential correlation of the Hirsch index with burnout symptoms separate from demographic and job-specific characteristics. A higher Hirsch index was related to lower emotional exhaustion scores, but did not explain, at least not statistically, the impact of being early in a professor career on burnout. Whether a low Hirsch index causes extra stress, or a high Hirsch index is a protective factor is a semantic, or even philosophical issue. In terms of career chances in academia, the Hirsch index may be a stressor for youngsters, but could also be reassuring for seniors. Furthermore the Hirsch index is correlated to personal accomplishment and all three subscales of engagement. Apparently, medical professors with a high Hirsch index feel they are more capable, have more vigour and dedication, and are more absorbed in their work.

Finally, engagement correlates moderately with burnout subscale scores. The interpretation of these correlations is hampered by the likelihood that the causality is bidirectional: engagement may protect against burnout, and burnout can severely compromise engagement. There may exist independent effects, but only longitudinal research can address this.

### Strengths and limitations

The strength of this study is that the survey was nationwide, addressing all medical professors in the country. Furthermore, burnout domain subscores were analysed on their natural, continuous scale, avoiding the loss of power associated with (arbitrary) dichotomisation of burnout symptomatology. In this respect, the topic of our study is more the propensity for developing burnout, rather than qualifying for any formal definition of the disorder.

A number of limitations also need to be addressed. First, we cannot rule out response bias. The survey completion rate was 36%, which is comparable to similar types of online questionnaires [28]. Although response bias is difficult to investigate, it is interesting to speculate in which direction it would occur. We think that response bias in our study may in fact have been bidirectional. Those experiencing more burnout symptoms could either preferentially participate (identification with the topic) or be reluctant to do so, caused by a sense of lack of time and task overload. To assure the representativeness of the sample we investigated the distribution of age and gender among all professors in the Netherlands. This population was representative as +/- 17% of the medical professors in the Netherlands is female (our sample 21%, http://www.stichtingdebeauvoir.nl/wp-content/uploads/Monitor_Vrouwelijke_Hoogleraren_2012.pdf) and the average age of professors in the Netherlands in another study including 1256 professors, was comparable with our mean ages [29]. This supports representativeness of our study population.

Since all medical professors in the Netherlands were invited for participation, the responders are not a sample from a sample but a sample of total study population. This further supports the representativeness of the study population. The population of medical professors is, obviously, heterogeneous. In the Netherlands, most have, at least formally, a part-time appointment as professor. All are more intensively involved in management, research and educational activities than regular physicians, but the degree to which this is the case may vary. Importantly, all professors in the Netherlands spend at least 1 to 2 days on patient care in view of registration legislation.

There may also be a taboo on burnout, causing respondents to downplay the severity and personal impact of burnout, despite the fact that anonymity was guaranteed. Another potential limitation could be the use of an online questionnaire for such a sensitive issue. However, the validity of online questionnaires is probably similar to ‘live’ questionnaires. [30] The timing of the study (September-October) could also have influenced the results and possibly attenuate burnout symptom scores, since national holidays are held in July and August, and academic work normally starts in the beginning of September.

A final important issue is the risk of framing: creating an atmosphere which stimulates ’positive answers’ depending on how the topic is introduced, how the questions are phrased, etc. To limit this risk, the invitation e-mail did not contain words such as ‘burnout’, but was phrased using more neutral words as ‘work engagement’ and ‘job satisfaction’ The Maslach Burnout Inventory is also constructed to reduce this risk of framing by including positive questions in the domain of personal accomplishment, which improves psychometric properties [31].

Maslach’s definition of burnout was originally a division of a sample into equal thirds and cut-off values were not mentioned. Burnout as a domain is most often defined as being above cut off on at least two dimensions (high emotional exhaustion and depersonalisation or high emotional exhaustion and low personal accomplishment). Since the Dutch bureau of statistics provides cutoff values for emotional exhaustion only, emotional exhaustion was, with possible limitation, chosen as a core feature of burnout. Furthermore since our study population consisted solely of ambitious and highly skilled medical professors the degree of burnout on the personal accomplishment domain was extremely low and was therefore considered not to be an appropriate feature of measuring burnout in this population.

Therefore we chose not to compare with other thresholds since in different research different thresholds are used and is therefore ambiguous and inconclusive [32, 33].

We also used cutoff values of the Dutch national Central Bureau of Statistics to allow a comparison with other Dutch professionals. In national samples of the total working force in the Netherlands, 11-14% meet the criteria for burnout. In another sample among Dutch doctors in residency training programs, the percentage was 41%, using the same definition as we did. However, the CBS assesses burnout using 5 statements from the emotional exhaustion scale to define moderate or severe burnout. Hence, these comparisons suggests that being a resident is be more stressful than being a professor, and that both are more prone to burn-out than the general Dutch working population.

Finally, the fact that we did not include other potential burnout determinants such as weekly work hours or work-home conflicts precludes more detailed analyses of the wider spectrum of determinants of burnout among this group.

## Conclusion

We conclude that emotional exhaustion is common among Dutch medical professors, and are determined by several factors, all related to being in an early stage of their professional career. Further research should focus on the impact of burnout on both the personal level, as well as on the level of professional performance in the clinical, educational and scientific domains. In future studies, potential preventive strategies should be addressed.
